# Stress and Occupational Coping among Brazilian Nurses in Critical Care Units during the COVID-19 Pandemic

**DOI:** 10.3390/healthcare12060613

**Published:** 2024-03-08

**Authors:** Silmara Meneguin, Camila Fernandes Pollo, Amanda Vitória Zorzi Segalla, Fary Jaqueline Fortaleza Generoso, Aniele de Leo, Cesar de Oliveira

**Affiliations:** 1 Department of Nursing, Botucatu Medical School, São Paulo State University, São Paulo 18618-970, Brazil; s.meneguin@unesp.br (S.M.); avzsegalla@gmail.com (A.V.Z.S.); fary.generoso@unesp.br (F.J.F.G.); aniele.fernanda@unesp.br (A.d.L.); 2 Department of Epidemiology & Public Health, University College London, London WC1E 6BT, UK

**Keywords:** coping, coping strategies, occupational stress, COVID-19, nursing staff

## Abstract

Objective: To investigate the effects of sociodemographic and working condition variables, as well as the coping strategies used by nurses, on their occupational stress during the COVID-19 pandemic. Methods: A cross-sectional study was conducted with 104 nurses who worked in intensive and emergency care at a public hospital in the state of São Paulo, Brazil. Data collection was performed in person and online using a questionnaire assessing sociodemographic and occupational characteristics, the Nursing Stress Inventory, and the Occupational Coping Scale. Results: The participants had a high level of stress (median = 132), especially in the ‘interpersonal relations’ domain (median = 63), and made little use of occupational coping strategies (median = 87). Income (*p* = 0.027), work shift (*p* = 0.028), being on leave from work (*p* = 0.020), number of hospitals with employment ties (*p* = 0.001), and relationship with management were independently associated with the levels of stress among the nurses. Conclusion: In the present study, the high levels of stress among nurses were influenced by financial and work-related factors as well as interpersonal relationships. No significant association was found between stress among the nurses and the use of occupational coping strategies.

## 1. Introduction

In late 2019, the first cases of COVID-19 caused by the novel coronavirus of severe acute respiratory syndrome 2 (SARS-CoV-2) were reported in Wuhan, China. At the beginning of 2020, the World Health Organization declared the disease a global health emergency [1,2].

As of August 2022, there have been more than 599 million confirmed cases of COVID-19 worldwide, with approximately 6.4 million deaths attributed to the disease. In Brazil alone, there have been 34 million confirmed cases and 683 thousand deaths [3]. The highly contagious nature of SARS-Cov-2 is considered to be one of the main reasons for the significant increase in the number of COVID-19-related deaths. As a result, many countries have implemented restrictive measures such as social distancing, confinement, and quarantine to try and slow the spread of the virus [4,5].

The outbreak of the COVID-19 pandemic has been considered a traumatic experience that has devastated all aspects of human life. It has aggravated mental health issues and caused distress due to the occurrence of the unknown infection in many fields [6,7]. The uncertainty and unpredictability of the situation have contributed to exacerbating psychological problems and stress during the pandemic [8].

Epidemics and pandemics can have negative impacts on the psychological well-being of healthcare professionals, leading to stress, depression, anxiety, insomnia, fear, stigma, and exhaustion [9].

Stress is the psychological result of an imbalance between perceptions of external desires and the internal resources available for satisfying such desires. Stress is considered a prevalent condition of the 21st century, affecting human beings in different ways and different contexts, and accounts for 31% of adverse health conditions and absenteeism from work among healthcare providers [10].

Occupational stress, which many individuals face in the workplace, results from a worker’s incapacity to meet work expectations, and has negative physical and psychological repercussions [8,11,12]. In recent years, occupational stress and associated outcomes have become a global health problem that poses a challenge for healthcare institutions [13]. Stressful situations are very frequent in the practice of nursing, especially in emergency wards, due to the multiple difficulties faced, such as the management of critical medical situations, the provision of care for severely injured patients, the frequent occurrences of death and trauma, the overcrowded environments, and the interrupted circadian rhythms due to the working shift [14]. Furthermore, nurses are the most frequent victims of physical attacks in psychiatric intensive care units [15].

During the peak of the COVID-19 outbreak in Brazil, there was a shortage of personal protective equipment (such as masks, gowns, gloves, and caps) for healthcare workers. This, combined with the excessive workload, resulted in various stressors that impacted the mental health of nursing staff. As a consequence, the quality of work performed by these workers and their quality of life were also negatively affected [16]. A study conducted with 516 nurses and employees of emergency services at a hospital in Iran found that up to 65% had mild to moderate occupational stress during the pandemic, demonstrating that COVID-19 increased work demands, diminished resources, and contributed to the increase in stress levels among these professionals [17].

Due to the inevitability of some stressors in the nursing profession and the need to prevent the negative psychological and behavioural effects of these factors on nursing staff, the use of effective strategies to assist individuals in dealing with stress is fundamental. Such strategies implemented in the workplace constitute what is denominated ‘occupational coping’ [18].

The term coping refers to a set of behavioural and cognitive actions used to face home or work environments with the aim of diminishing the harm caused by stressful situations. Therefore, coping can be understood as a strategy for minimising the effects of occupational stress [19]. A study conducted in Spain with 421 nurses from 39 provinces of the country at the onset of the pandemic found that nurses with insufficient preparation and those with high levels of fear of contagion did not employ adequate coping strategies [20].

However, the relationship between stress and occupational coping during the pandemic is still a topic that has been scarcely explored in the literature, despite its importance. Thus, this investigation could contribute to the adoption of more comprehensive strategies for the prevention of the occupational stress during pandemics in the hospital setting as well as for the reorganisation of care.

This study aims to investigate the effects of sociodemographic and working condition variables, as well as the coping strategies used by nurses, on their occupational stress. Due to the limited research in this area, the study seeks to answer the following questions: What was the level of stress experienced by frontline nurses in Brazil during the pandemic? Did the participants use coping strategies, and if so, what were they? Are sociodemographic and labour variables associated with stress during the pandemic? Is there a relationship between occupational coping and stress? Therefore, the present study aimed to investigate stress among Brazilian nurses in critical care units and its association with occupational coping strategies, and sociodemographic and work variables during the COVID-19 pandemic.

## 2. Methods

### 2.1. Study Design

A descriptive, exploratory, cross-sectional study was conducted with a quantitative approach.

### 2.2. Study Setting

The study was carried out at the intensive care units (ICUs) and emergency units of a public hospital that is a regional reference for cases of COVID-19 in the state of São Paulo, Brazil. This hospital has 415 beds, and during the pandemic, 30 adult ICU beds were opened for COVID-19 patients.

### 2.3. Study Population

Female and male nurses who met the following inclusion criteria were considered eligible for the study: those who were working in adult COVID-19 ICUs and/or emergency units and agreed to participate in the study by signing a statement of informed consent. Individuals who exclusively exercised administrative duties, those on vacation or on leave from work, and those without the self-reported emotional strength to participate in the interview were excluded.

#### Calculation of Sample Size

Considering the prevalence of burnout among nursing staff to be 46%, with a 95% confidence level and 10% margin of error, the minimum sample size was determined to comprise 94 participants. The following formula was used:

$$n={\left(\frac{z_{\alpha /2}\sqrt{p(1-p)}}{\varepsilon }\right)}^{2},$$

where

*z_α_*_/2_ = 95% quantile of the normal distribution (1.96);
*p* = stress prevalence (46%) [21];
*ε* = margin of error (10%).

### 2.4. Data Collection

Data collection was performed in person and online, using Google Forms^®^, at the hospital in the period from July 2020 to September 2021. The average response time was 15 min. The reason for the relatively short data collection period was due to time constraints since other postgraduate research projects were running at the same time that needed to be concluded.

Three instruments were used for data collection. The first was a questionnaire assessing sociodemographic characteristics. The second was the Portuguese version of the Nursing Stress Inventory, and the third was the Occupational Coping Scale. 

The Nursing Stress Inventory (NSI) was developed at the end of the 1980s for the assessment of stress levels in nurses and was validated for use in Brazil in the year 2000 [22,23]. The NSI comprises 38 items with response options on a Likert scale ranging from 1 to 5 points (1 = never; 2 = rarely; 3 = sometimes; 4 = often; and 5 = always). The total ranges from 38 to 190 points, with higher scores denoting a greater likelihood of developing stress in the workplace [23].

The NSI addresses situations in the healthcare setting that can cause stress, distributed across three domains: interpersonal factors, stressful career roles, and factors intrinsic to work. Interpersonal factors refer to how the respondent relates to other healthcare providers, patients, and their families, as well as students and the respondent’s own family. Stressful career roles include situations such as a lack of recognition and autonomy, impotence regarding the execution of tasks, and other aspects related to the institution and physical environment. Factors intrinsic to work involve working hours, issues with materials at work, and factors related to salary [23]. 

The Occupational Coping Scale (OCS) is used to measure coping strategies for handling stress in the work environment. The scale was developed by Latak in 1986 [24] and was translated, adapted, and validated for use in Brazil in 2003. This scale comprises 29 items scored based on the sum of affirmative statements (1 = ‘I never do this’; 2 = ‘I rarely do this’; 3 = ‘I sometimes do this’; 4 = ‘I often do this’; and 5 = ‘I always do this’). The total ranges from 29 to 145 points, with higher scoring denoting greater ease in developing strategies for dealing with stressful situations [25]. The scale has three domains: control, which regards self-control strategies developed for stressful situations; escape, which regards measures for escaping stressful situations; and the management of symptoms, which regards strategies for dealing with widely accepted symptoms of stress [25].

### 2.5. Statistical Analysis

Considering the prevalence of stress among nurses to be 46%, with a 95% confidence level and 10% margin of error, the minimum sample size was determined to comprise 94 participants.

All variables were first analysed descriptively. The Shapiro–Wilk test was used to check the normality of the data. A median (p25–p75) was used to calculate NSI and OCS scores. The internal consistency of the scale and its dimensions was evaluated using Cronbach’s alpha coefficients, with values greater than 0.70 considered acceptable [26].

For the correlation analysis between the instruments, the Spearman coefficient was used, considering the following values for interpretation: 0.00 to 0.19 as a very weak correlation, 0.20 to 0.39 as a weak correlation, 0.40 to 0.59 as a moderate correlation, 0.60 to 0.79 as a strong correlation, and 0.80 to 1.00 as a very strong correlation [27]. Generalised linear regression models were run to determine differences in NSI and OCS scores according to sociodemographic and work-related factors (stepwise model). All analyses were performed using the IBM SPSS program, version 22, with the significance level set at 5% (*p* < 0.05) [28].

The checklist of the Strengthening the Reporting of Observational Studies in Epidemiology (STROBE statement) was followed to ensure the quality of the study. This study received approval from the Human Research Ethics Committee of the Botucatu School of Medicine.

## 3. Results

One hundred and four nurses participated in the present study. Most were young (median age: 31 years), were women (n = 83; 79.8%), had a spouse/partner (n = 65; 62.5%), did not have children (n = 68; 65.4%), followed the Catholic faith (n = 64; 61.5%), worked the day shift (n = 65; 62.5%) at only one hospital (n = 76; 73%), and had a specialisation in the field (n = 49; 47.1%). Nearly half of the sample (48%) had a monthly income of USD 1000 or more. Fifty-eight had been on leave from work, mainly due to suspected or confirmed COVID-19 infection (n = 46; 44.2%) (Table 1). Among the stressful situations described, the most cited referred to the work environment, which was mentioned by 54 participants.

Table 2 displays the median (25th–75th percentile) of the NSI and OCS scores, and the respective domain scores. The ‘interpersonal relations’ domain of the NSI had the highest score for the development of higher levels of stress. On the OCS, the ‘control’ domain had the highest score with regard to the coping techniques adopted.

Table 3 displays the results of the multivariate analyses performed using the generalised linear model for the NSI. Not having a partner (*p* = 0.033), emergencies (*p* = 0.041), and relationship with management (*p* = 0.028) were positively associated with stress in the ‘interpersonal relations’ domain.

Positive associations were found between stress in the ‘stressful career roles’ domain and monthly income more than USD 1000 (*p* = 0.027), relationship with management (*p* = 0.026), and work environment (*p* < 0.001). However, working the day shift (*p* = 0.028), having been on leave (*p* = 0.020), and number of hospitals with employment ties (two: *p* = 0.010; three: *p* = 0.001) were associated with a reduction in stress in this domain.

Stress in the ‘factors intrinsic to work’ domain was negatively associated with the number of hospitals with employment ties (*p* = 0.008). In contrast, positive associations were found with death of the patient (*p* = 0.047), relationship with management (*p* = 0.010), and work environment (*p* < 0.001).

Table 4 displays Spearman’s correlation matrix for total NSI and OCS scores, and the respective domain scores. No correlation was found between the two instruments.

Cronbach’s alpha of the NSI was 0.72 and that of the OCS was 0.75; both are generally suitable to assess accuracy with.

## 4. Discussion

This study investigated the level of stress among Brazilian nurses and its association with occupational coping strategies in critical care units during the COVID-19 pandemic. Many nurses suffered from psychological distress and stress during the COVID-19 pandemic, with an excessive workload causing stress.

Concerning the sociodemographic data, most of the participants in the present study were young (median age: 31 years), were women (79.8%), had a partner (62.5%), received a monthly wage higher than USD 1000 (48%), and had no children (65.4%). These results are in agreement with data reported from other Brazilian studies, in which the median age of the nursing team was 32 years, 79.8% were women, 52% were married, and the majority received between 9 and 12 times the monthly minimum wage (~USD 244) [29].

In total, 58 (58%) nurses had been on leave from work, 46 (79.4%) of whom had a suspected or confirmed infection diagnosis for COVID-19. Excessive workloads, job instability, difficult access to personal protective equipment, and other factors related to the daily work routine resulted in the vulnerability of healthcare providers. Thus, these frontline workers became more susceptible to contamination, resulting in thousands being on leave due to COVID-19 [30,31]. The work organisation changed at the onset of the pandemic, with an increase in workload and the need to work extra hours during the shift, imposing an exhausting pace [32]. A study developed in China reported that prevention and health protection measures should be adopted for healthcare providers who work on the frontline, with a reduction in the work shift to less than 10 h per day. Moreover, rest periods are needed during the shift to diminish physical and psychological stress [33,34].

In the actual investigation, the level of stress was high among the participants, especially in the ‘interpersonal relations’ domain. Previous evidence showed that nurses who worked on the frontline were more prone to higher levels of stress due to the conflicts and ambiguities of roles that they were expected to develop [35,36]. This occupational exposure can have negative impacts on psychological, emotional, and social aspects, as described in an integrative review of the literature conducted in 2022 [37]. A study carried out in Egypt showed that working with patients with COVID-19 was the main cause of stress [38]. However, these results differ in some countries, such as Turkey, where the level of stress experienced during the pandemic by 262 nurses was moderate.

Concerning the use of coping strategies, coping was moderate in general, and the control domain of coping strategies was the most cited by study participants. This finding can be explained by the pandemic period, which required greater control from professionals to face a situation until then unknown to everyone. Although this result shows that participants used coping strategies appropriate to this situation and is corroborated by a study that preceded the pandemic [39], we do not intend to carry out future explorations of this finding as the current situation is very different from the period in which the research was carried out, which required professionals to remain calm, work under pressure, and control highly stressful situations, with inadequate power.

A literature review conducted in 2021 reported the consensus between all studies on the psychological effects of the pandemic on nursing staff as well as the entire population and the coping strategies adopted by workers. In contrast, previous studies demonstrated that healthcare providers used problem-centred and emotion-centred strategies to manage stress associated with the COVID-19 pandemic. Such studies also demonstrated that the conscious search for solutions to problems favours decision-making and effective coping strategies, improves patient care and the quality of life of the staff, and enhances job satisfaction [40,41].

In the multivariate analysis using the generalised linear model of the NSI, significant associations were found between the ‘interpersonal relations’ domain and not having a partner, emergencies in the work environment, and the relationship with management. Similar findings were reported in a study conducted with nurses in southern Brazil, which recognised that interpersonal relations cause the greatest friction in day-to-day work [42]. Another study conducted with 207 nurses in Korea during the pandemic confirmed these findings and revealed that conflicts with physicians, patients, and caregivers as well as [43] other interpersonal relations contributed to the development of occupational stress [44].

In the present study, stress was also associated with the ‘stressful career roles’ domain. Participants in situations involving a lack of definition of the role of nurses due to the unpredictability of tasks, a lack of recognition, autonomy, and other aspects that affect the physical and personal environment of the institutions, nurses with higher wages, and those who experience situations of stress in the relationship with management and the work environment scored higher for developing stress in this domain.

In contrast, nurses who needed to go on leave, those who worked the day shift, and those who worked in more than one hospital had lower scores for stress in this domain. These findings diverge from data reported in a study conducted at urgent care units in the state of São Paulo, in which nurses who worked the day shift were more likely to have positive scores for the development of stress [45]. In the ‘factors intrinsic to work’ domain, a significant increase in stress levels was found in nurses with experience involving the death of patients. Although death is a natural process, the feelings it provokes cause an imbalance of mental faculties, especially among intensive care staff, who experience numerous situations of loss of their patients [46]. This was confirmed in a study conducted in Spain that used a post-traumatic stress scale and found that nurses had a tendency to develop stress when caring for patients with COVID-19 and experiencing the death of such patients in comparison with those who did not undergo such situations [47].

Stressful conditions in the workplace and the relationship with management were positively associated with developing stress in this domain. Management is a relevant point in the work process and plays an important role, especially in situations such as the COVID-19 pandemic. Leaders should seek strategies to keep the staff protected from stressful factors and mental health problems [48]. However, a lack of tact when dealing with such contexts can aggravate stress among the members of the team, which may explain the increase in stress among the participants of the present study for all domains related to the relationship with management. 

Lastly, no correlation was found between the assessment instruments and respective domains, confirming that there was no relationship between them.

The present study has limitations that should be considered. The cross-sectional design with interviews restricted to a single moment in time may not have been sufficient to portray the magnitude of the changes imposed by the pandemic. This may impact the generalisability of our findings. The considerable predominance of women in the sample and the short time working in the critical care unit could also be considered sources of bias in this study. The data collection period from July 2020 to September 2021 may not fully capture the pandemic’s evolving impact on healthcare professionals. This period coincided with ongoing research projects that needed to be concluded. Furthermore, our sample may have been adequately powered to assess the overall level of stress among nurses during the pandemic, but analyses of correlates and predictors are entirely exploratory.

## 5. Implications for Practice

Given the relevant influence of the pandemic on the mental health of nurses around the world, a study of stress during this period is highly relevant and can provide important insights into dealing with the psychosocial impacts left by this period. Furthermore, the identification of contributing factors during the pandemic allows institutions to better organise themselves to develop protective strategies against work stress, which should include a reduction in weekly working hours, decent working conditions, self-care practices, and psychosocial support.

Better working conditions must be provided so that nurses can have, in addition to rest between work shifts, a mental health monitoring program. However, future research is needed to assess whether or not these protective measures were implemented after the pandemic to reduce stress levels within hospitals.

## 6. Conclusions

The main findings of the present study indicate that the level of stress was high among the participants, especially in the ‘interpersonal relations’ domain. Income, marital status, relationship with management, and work environment were independently associated with stress levels. In contrast, the number of hospitals with employment ties was negatively associated with this construct. The use of coping strategies was moderate in general, but low in terms of the ‘escape’ and ‘management of symptoms’ domains.

Further studies should be developed with a longitudinal design to contribute to improvements in the working conditions of nursing staff. A broader approach to this issue is needed due to the relevance to this context, as the work of nurses in the direct care of critical patients is indispensable during the COVID-19 pandemic.

## Figures and Tables

**Table 1:** Sociodemographic and work-related characteristics and stressful situations, 2022.

Variable	n (%)
Age (years)	
Median	31
(25th–75th percentile)	(27–36.5)
Work setting	
Emergency room	50 (48.0)
ICU *	54 (52.0)
Time working in profession (months)	
Median	44
(25th–75th percentile)	(20–120)
Time working in unit (months)	
Median	14
(25th–75th percentile)	(6–36)
Hours worked per week during pandemic	
Median	40
(25th–75th percentile)	(40–60)
Gender	
Female	83 (79.8)
Male	21 (20.2)
Marital status	
With partner	65 (62.5)
Without partner	38 (36.5)
Did not answer	1 (0.9)
Children	
No	68 (65.4)
Yes	35 (33.7)
Did not answer	1 (0.9)
Years of schooling	
Undergraduate university/college education	41 (39.4)
Specialisation	49 (47.1)
Master’s degree	14 (13.5)
Monthly income in USD	
USD 200 to 600	16 (15.4)
USD 601 to 1000	38 (36.5)
More than USD 1000	50 (48.1)
Religion	
Catholic	64 (61.5)
Non-Catholic	38 (36.5)
Did not answer	2 (1.9)
Work shift	
Day	65 (62.5)
Night	39 (37.5)
Had been on leave	
No	46 (44.2)
Yes	58 (55.8)
Reason for being on leave	
COVID-19	23 (22.1)
Other	12 (11.5)
Suspected COVID-19	23 (22.1)
Stressful situations	
Emergency care	37 (35.5)
Death of patient	47 (45.1)
Relationship with management	23 (22.1)
Relationship with team/physicians	42 (40.3)
Work environment	54 (51.9)
Family relations	17 (16.3)
Work process	31 (29.8)

* ICU = intensive care unit.

**Table 2:** Distribution of the median (25th–75th percentile) of NSI, OCS, and respective domain scores, 2022.

Variable	Median	25th–75th Percentile
NSI *		
Interpersonal relations	63	(49.5–71)
Stressful career roles	34	(28–39)
Factors intrinsic to work	34	(29–39.5)
Total	132.0	(111–146)
OCS **		
Control	43	(39–45.5)
Escape	22	(18.5–25)
Management of symptoms	22	(18–26)
Total	87	(78–94.5)

* NSI = Nursing Stress Inventory; ** OCS = Occupational Coping Scale.

**Table 3:** β coefficients of generalised linear model for NSI domains according to sociodemographic and work-related variables, 2022.

Exploratory Variables	Interpersonal Relationships		Stressful Career Roles		Factors Intrinsic to Work	
	β Coefficient	*p*	β Coefficient	*p*	β Coefficient	*p*
Work setting						
Ref. ICU	4.48887	0.135	0.15638	0.923	1.3815	0.319
Age	0.19139	0.482	−0.01526	0.918	0.0897	0.478
Sex						
Ref. Male	1.36646	0.704	3.59876	0.069	1.4309	0.391
Marital status						
Ref. Without partner	7. 03329	0.033	1.41858	0.424	0.1550	0.918
Children						
Ref. With children	6.18641	0.127	0.71302	0.745	−1.8683	0.319
Schooling						
Ref. Specialisation	4.52788	0.130	2.54366	0.119	1.9325	0.163
Monthly income						
Ref. > USD 1000	1.43005	0.780	6.28597	0.027	3.5963	0.132
Work shift						
Ref. Morning/Afternoon	−10.13155	0.074	−6.81264	0.028	−4.3430	0.098
Time in profession (months)	−0.02706	0.322	0.00746	0.616	−0.0134	0.290
Time in unit (months)	−0.00755	0.887	0.02230	0.444	−5.90 × 10^−5^	0.998
Having been on leave						
Ref. Yes	3.96584	0.181	−3.81176	0.020	−1.6182	0.239
Number of hospitals with employment ties						
Ref. 2 vs. 1	−7.31307	0.121	−6.69876	0.010	−5.9057	0.008
Ref. 3 vs. 1	17.02152	0.262	−27.90611	0.001	−7.5330	0.284
Situations considered stressful						
Emergency care						
Ref. Yes	6.45727	0.041	1.52763	0.370	2.3597	0.106
Death of patient						
Ref. Yes	3.74349	0.197	1.48891	0.345	2.6866	0.047
Relationship with management						
Ref. Yes	8.28284	0.028	4.56538	0.026	4.5181	0.010
Work environment						
Ref. Yes	5.56345	0.091	6.92701	<0.001	6.0595	<0.001
Occupational coping	0.59865	0.644	2.10009	0.130	−0.27255	0.860

**Table 4:** Spearman’s correlation matrix for total NSI and OCS scores, and respective domain scores, 2022.

		Total Stress	Interpersonal	Career Stressor	Intrinsic Work	Total OCS	Symptoms	Control	Escape
Total stress	Spearman’s rho *p*-value								
Interpersonal	Spearman’s rho *p*-value	0.804 <0.001							
Career stressor	Spearman’s rho *p*-value	0.754 <0.001	0.312 0.001						
Intrinsic work	Spearman’s rho *p*-value	0.856 <0.001	0.498 <0.001	0.712 <0.001					
Total OCS	Spearman’s rho *p*-value	0.110 0.260	0.174 0.072	−0.004 0.966	0.033 0.736				
Symptoms	Spearman’s rho *p*-value	−0.060 0.542	0.047 0.632	−0.140 0.150	−0.096 0.326	0.767 <0.001			
Control	Spearman’s rho *p*-value	0.172 0.077	0.265 0.006	0.004 0.970	0.081 0.406	0.494 <0.001	0.118 0.226		
Escape	Spearman’s rho *p*-value	0.091 0.353	0.042 0.666	0.114 0.244	0.061 0.529	0.692 <0.001	0.303 0.002	0.121 0.216	

## Data Availability

The data that support the findings of this study are available on request from the corresponding author. The data are not publicly available due to restrictions, e.g., because they contain information that compromise the privacy of research participants. All listed authors meet the authorship criteria and all authors agree with the content of the manuscript.
